# Robot‐assisted Minimally‐invasive Internal Fixation of Pelvic Ring Injuries: A Single‐center Experience

**DOI:** 10.1111/os.12423

**Published:** 2019-02-03

**Authors:** Hua‐shui Liu, Sheng‐jun Duan, Fu‐zhen Xin, Zhen Zhang, Xue‐guang Wang, Shi‐dong Liu

**Affiliations:** ^1^ Department of Traumatic Orthopaedics Affiliated Jinan Third Hospital of Jining Medical University Jinan China

**Keywords:** Fracture fixation, Internal, Minimally‐invasive surgery, Pelvis, Robotics

## Abstract

**Objective:**

To investigate the indications, surgical strategy and techniques, safety, and efficacy of robot‐assisted minimally‐invasive internal fixation of pelvic ring injuries.

**Methods:**

The clinical data of 86 patients with anterior and posterior pelvic ring injuries who underwent robot‐assisted minimally‐invasive internal fixation were retrospectively analyzed. The patients included 57 men and 29 women aged between 22 and 75 years, with an average age of (40.2 ± 13.6) years. According to the Tile classification, there were 5 (5.8%) type A2, 48 (55.8%) type B, and 33 (38.4%) type C fractures. The surgical plans were formulated based on the injury type of the pelvic ring, the effectiveness of the reduction, and the integrity of the osseous channel. Posterior pelvic ring injuries were treated with robot‐assisted percutaneous cannulated screw fixation of the sacroiliac joint. Anterior pelvic ring injuries were treated with robot‐assisted percutaneous cannulated screw fixation of the pubic ramus, INFIX fixation, or a “hybrid” fixation. The surgical complications and the efficacy of the surgical treatments were analyzed.

**Results:**

A total of 274 screws were inserted with robotic assistance, of which 262 screws were successfully inserted to a satisfactory position on the first attempt. The number of screws placed per person was 3.2 on average, and the average operation time was 175 min (35–280 min). Fluoroscopies were performed an average of 29.1 times (range, 9–63 times), and it took 6.1 s to place each screw. There were 13 unsatisfactory guiding needle placements during the surgeries, among 7 of which cutting or penetration of the cortex was re‐planned until satisfactory insertions; 1 penetrated the pubic cortex, causing hemorrhage of the “crown of death,” and was changed to “hybrid surgery”. The robot‐assisted surgical wounds all healed by primary intention with satisfactory position and precision of screw insertions. All patients were followed up for 3–6 months, with an average of 4.2 months. There were two postoperative fixation failures, in which both patients had separated symphysis pubes after hybrid surgery. The average Majeed score at the last follow‐up was 92.4 points.

**Conclusions:**

Robot‐assisted surgery is accurate and minimally invasive, with a high success rate for one‐time screw placement and satisfactory clinical results. The indications and surgical strategy should be rigorously selected, the level of surgical techniques mastered, and the operating procedures standardized, all of which may help to prevent surgical complications. Robot‐assisted surgery provides a novel modality for the minimally‐invasive treatment of pelvic ring injuries.

Pelvic fractures are often caused by high‐energy trauma resulting in injuries of both anterior and posterior pelvic rings. Simultaneous anterior and posterior pelvic ring fixations are often necessary to maintain the stability of the pelvic ring and to achieve similar biomechanical properties to a normal pelvis^1^.

The fixation methods for posterior and anterior pelvic ring injuries include external fixation, open reduction and internal fixation with plates, and minimally‐invasive percutaneous sacroiliac screw fixation and pubic ramus screw fixation^2^, ^3^. The traditional open reduction and internal plate fixation achieves the best anatomic reduction and provides strong fixation. However, its drawbacks include severe surgical trauma, more bleeding, and common damage to nearby critical vessels and nerves, which will influence postoperative recovery.

Minimally‐invasive surgery is a current trend in modern medicine. Robot‐centered technology for precision orthopaedic treatments has become one of the major directions in the development of surgical techniques^4^, ^5^, ^6^. The concept of surgery for pelvic ring injuries is also continuously being updated. The surgical approach has gradually changed from the conventional open reduction and internal fixation to minimally‐invasive screw internal fixation^7^, ^8^, ^9^.

Minimally‐invasive fixation using combined sacroiliac and anterior column screw placements is an alternative surgical approach to the treatment of anterior and posterior pelvic ring fractures^10^, ^11^. X‐ray‐guided percutaneous sacroiliac screw fixation or pubic ramus screw fixation is a good surgical procedure for stabilizing the pelvic ring. This method demonstrates significantly less invasiveness and fewer complications, especially for pubic ramus screw fixation of the anterior pelvic ring. Biomechanical studies have shown that sacroiliac screw fixation of the posterior pelvic ring exhibits reliable mechanical strength and can provide consistent pelvic stability^12^, ^13^. However, it is difficult to ensure that each screw is located in the best position under freehand fluoroscopic guidance alone. The accuracy of the insertions can vary, and the fluoroscopy procedure also increases radiation exposure, potentially leading to tissue damage among patients and medical personnel. Robot‐assisted or computer‐assisted 3D fluoroscopy‐based navigation is undoubtedly the best option for precise screw placements^6^, ^14^, ^15^.

China owns the complete intellectual property rights for the “TianJi” robotic system, which was independently developed as a “Key Research and Development of Digital Diagnosis and Treatment” project of the Ministry of Science and Technology in China for use during orthopaedic surgery procedures. The third‐generation orthopaedic surgery robot TiRobot represents the latest generation of versatile state‐of‐the‐art robot‐based navigation systems for orthopaedic surgery. This robotic system adopts a modularized, miniaturized, and generalized design^7^, and can assist physicians to accurately plan the positions, trajectories, and lengths of screw insertions using minimal X‐ray irradiation. It is a guide for physicians to complete fixation surgeries efficiently and safely. Compared with traditional surgery, this robotic system is simple to operate, precise with respect to positioning, is minimally invasive, requires short operative times, and causes minimal radiative damage^16^, ^17^, ^18^, ^19^. These properties are aligned with mainstream minimally‐invasive orthopaedic treatments domestically and abroad^8^, ^9^. Since this system was introduced in our hospital, we have performed robot‐assisted minimally‐invasive internal fixations to treat various types of pelvic ring injuries. The number of patients we have treated with this system is among the highest in the country, and, hence, we have accumulated considerable experience in operating the system.

We identified patients who underwent robot‐assisted minimally‐invasive internal fixations of pelvic fractures and analyzed the surgical plans selected for different types of pelvic ring injures. The purposes of this study were: (i) to investigate the indications and contraindications for robot‐assisted internal fixations of pelvic ring injures; (ii) to analyze the safety and efficacy of the surgery; and (iii) to discuss surgical strategy, techniques, precautions, and existing problems with robot‐assisted surgery for pelvic ring injures.

## Methods

### *Inclusion and Exclusion Criteria*

The inclusion criteria were: (i) closed, unstable pelvic ring injuries (Tile type B or C fractures), with or without fracture displacement, that could easily be treated with closed reduction (or limited open reduction), after which a bone tunnel was available for cannulated screw placements; (ii) patients with closed Tile type A2 fracture who could not tolerate bedridden or required early movement; and (iii) patients with fixable open Tile type B and C fractures that became closed fractures after initial treatment and were eligible for reduction and screw placements. Exclusion criteria were: (i) presence of severe open injuries or rupture of the abdominopelvic cavity and organs with wound contamination; (ii) unstable hemodynamics; (iii) tissues such as blood vessels and nerves in the robot‐planned trajectory that could not be avoided, or patients without a bone tunnel for cannulated screw placement after reduction, or patients with fractures in which effective screw fixation could not be achieved; (iv) hardware or patient factors resulting in poor image acquisition and failure to safely conduct procedure planning; (v) poor condition of local skin, or infection of the soft tissues at or around the screw insertion site; (vi) systemic diseases such as severe bleeding disorders, severe heart disease, and severe respiratory disease; and (vii) inability to tolerate anesthesia or surgery.

### *Patients’ Information*

This retrospective study reviewed a case series from November 2016 to May 2018. The study protocol was approved by our Institutional Review Board. Informed consent was obtained from all participants included in the study. A total of 86 patients with anterior and posterior pelvic ring injuries (including nine acetabular or iliac wing fractures) underwent robot‐assisted internal fixations at the author's (L. Huashui's) institution. The study population included 57 men and 29 women, with an average of 40.2 ± 13.6 years (range, 22–75 years). There were 71 closed fractures, 15 open fractures that became closed fractures after initial treatment, and 47 combined non‐severe injuries in other body locations. According to the Tile classification, there were 5 type A2, 48 type B, and 33 type C fractures. All operations were performed by the same team of surgeons who possessed adequate clinical experience in orthopaedic traumatology.

### *Preoperative Treatment*

After admission, the patients’ vital signs were monitored, venous access was established, and comprehensive and focused physical examinations were conducted. Patients were also given urethral catheterization and blood volume expansion therapy. Patients with unstable hemodynamics were treated with temporary pelvic external fixation. Patients with vertical instability were treated with lower extremity traction. Routine imaging of the pelvis was performed using X‐ray, CT scans, and 3D reconstructions. After initial treatment, follow‐up radiographs were obtained when the patients became hemodynamically stable. The surgical plans were formulated based on the injury type of the pelvic ring, the effectiveness of the reduction, and the availability of bone tunnels. Most surgeries were performed between days 3 and 14 (mean, 5.6 ± 2.6 days) after initial injury.

### *Surgical Equipment and Instrument*

The TiRobot system, the third generation TianJi robot for orthopaedic surgery (TINAVI Medical Technologies, Beijing, China), is composed of a main console, a robotic arm, surgical planning and controlling software, an optical tracking system, a main control workstation, and a navigation and positioning toolkit. Additional surgical equipment included a C‐arm X‐ray machine (Siemens, Germany), ϕ7.3‐mm cannulated screw and ϕ6.5‐mm pedicle screw systems (Tianjin Zhengtian Medical Instruments, China), and reconstruction locking plates (Shandong Weigao Orthopedic Device, China).

### *Surgical Strategy and Procedures*

The surgical strategy is presented in Table 1: isolated anterior pelvic ring injuries were treated with robot‐assisted percutaneous cannulated screw fixation of the pubic ramus; posterior pelvic ring injuries were treated with robot‐assisted percutaneous cannulated screw fixation of the sacroiliac joint; and complex anterior pelvic ring injuries (including symphysis pubis separation) were treated with cannulated pubic ramus screw fixation alone, INFIX fixation or “hybrid surgery” (open reduction and internal plate fixation). Among the combined injuries, 54 cases were initially treated for the anterior pelvic ring injuries followed by posterior pelvic ring injury treatments, and 27 cases were initially treated for the posterior pelvic ring injuries followed by anterior pelvic ring injury treatments.

**Table 1:** Pelvic ring injury type, surgical strategy, and surgical complications

Pelvic ring injury type	*n*	Surgical strategy	Surgical complications
Isolated anterior pelvic ring injury	5	Robot‐assisted percutaneous cannulated screw fixation of the pubic ramus	—
Posterior pelvic ring injury with symphysis pubis separation	6	“Hybrid surgery”: Robot‐assisted percutaneous cannulated screw fixation of the sacroiliac joint and open screw or plate fixation of the symphysis pubis	Fixation failure after screw loosened in one case of anterior pelvic ring Subcutaneous ecchymosis in one case
Posterior pelvic ring injury with unilateral anterior pelvic ring injury	32	Posterior pelvic ring: Robot‐assisted percutaneous cannulated screw fixation of the sacroiliac joint Anterior pelvic ring: Robot‐assisted percutaneous cannulated screw fixation of the pubic ramus	Two cases of penetration of pubic cortex and one case of hemorrhage of the “crown of death” One case of needle cutting and one case of penetration of the sacrum cortex
Posterior pelvic ring injury with bilateral anterior pelvic ring injury	11	Cannulated screw fixation alone: Robot‐assisted percutaneous cannulated screw fixation of the sacroiliac joint and pubic ramus	One case of needle cutting of the pubic ramus cortex
	19	Posterior pelvic ring: robot‐assisted percutaneous cannulated screw fixation of the sacroiliac joint Anterior pelvic ring: Manual or robot‐assisted pedicle screw placement combined with INFIX fixation	One case of needle cutting of the sacrum cortex One case of femoral cutaneous nerve injury during manual placement of the pedicle screw One case of failure of anterior pelvic ring fixation after removal of INFIX
	13	“Hybrid surgery”: robot‐assisted percutaneous sacroiliac screw fixation of the posterior pelvic ring combined with open reduction and internal fixation of the anterior pelvic ring	One case of needle cutting of the sacrum cortex One case of skin edge necrosis One case of wound (subcutaneous) infection
(Combined) acetabular fractures and/or iliac wing fractures	9	Robot‐assisted percutaneous cannulated screw fixation of anterior column Robot‐assisted screw fixation, or open reduction and internal plate fixation of iliac wing	One case of needle penetration of iliac wing One case of subcutaneous hematoma

INFIX, anterior subcutaneous pelvic fixation.

During the surgical procedures, patients were administered general anesthesia with tracheal intubation after being placed in the supine position. The surfaces were sterilized by routine disinfection and draping. A tracker was fixed on the contralateral anterior superior iliac spine. Then, a sterile working environment for the robotic arm was established by assembling and fixing the robot tracker and the sterile protective sleeve. After installing the calibrator for the robot tracker, the surgeon moved to an appropriate position on the ipsilateral side near the operating bed to initiate the support systems and to lock the position of the robot. Images of the pelvic fractures corresponding to each surgical procedure were taken using a fluoroscope fixed with a calibrator. The fluoroscopic images were then transmitted to the robotic planning system. Based on the patient's anatomic features and the fracture conditions, the surgeon designed the instructions using the planning system and completed the simulation of the cannulated screw placement on the images. After a plan was established, the robotic arm began to move according to the instructions. The guidance in the pre‐planned trajectory was completed outside the body using guidewires and sleeves. The skin was incised, and the subcutaneous layer was separated. The sleeve was placed onto the bone surface, and the trajectory was recalibrated. The guiding needle was inserted along the trajectory and the cannulated screws were then inserted along the needle. The positioning of the cannulated screws was verified by fluoroscopy. The wound was subsequently rinsed, the subcutaneous layer and skin were sutured, and the surgery was completed.

### *Postoperative Rehabilitation and Follow‐up*

Prophylactic antibiotics were used for 48 h after surgery. Low‐molecular weight heparin calcium was used for 4 weeks for prevention of deep vein thrombosis treatment. Follow‐up imaging of X‐ray and CT scan were performed 72 h after fixation. Postoperative rehabilitation training was conducted according to the patient's injury severity, fracture type, and methods used for internal fixation. For patients with isolated pelvic injuries and satisfactory internal fixations, stretching exercises of the hip and knee joints could be performed after recovering from anesthesia. Two days after surgery, the patients were allowed to perform moderate exercises that involved body turning. The exercise intensity could be gradually increased for patients with combined injuries. Patients with an isolated anterior pelvic ring injury were allowed to get out of bed and start walking with the help of crutches at 1 week after surgery. The timing for patients with combined anterior and posterior pelvic ring injuries to get out of bed and perform weight‐bearing activities was based on the severity of their injuries and the fracture healing process. Generally, these patients could begin walking with two crutches 2–4 weeks after surgery, walk with one crutch and lean on the contralateral leg in 5–7 weeks, and then walk with full weight‐bearing in 8–10 weeks. The process for patients with severe injuries was delayed by 2 weeks.

The surgical complications, especially for the penetration accidents resulting from needle deviation, were recorded. All patients were available at a mean follow‐up of 4.2 months (3–6 months). The patients received monthly follow‐up pelvic radiographs until the fractures were healed. The position and accuracy of the robot‐assisted screw placements were evaluated according to the radiographic data of the last follow‐up. During the follow‐up period, patients were queried as to their daily activities, pain levels, standing difficulties, and walking distances to determine their tolerance to the screws inserted during the internal fixation procedure. Evidence for wound infection, screw loosening, and nerve injury was evaluated. Patients with nerve injuries were treated with neurotrophic drugs. The dressings were changed more frequently in patients with wound infections. Efficacies were evaluated according to the Majeed score.

## Results

### *General Results*

According to the type of pelvic ring injuries, 5 patients with isolated anterior pelvic ring injuries were treated with robot‐assisted percutaneous cannulated screw fixation of the pubic ramus, 6 patients with posterior pelvic ring injury combined with symphysis pubis separation were treated with robot‐assisted percutaneous cannulated sacroiliac screw fixation and open screw or plate fixation of the symphysis pubis, 32 patients with posterior pelvic ring injury combined with unilateral anterior pelvic ring injury were treated with robot‐assisted percutaneous cannulated sacroiliac screw fixation pubic ramus screw fixation, and 43 patients with posterior pelvic ring injury combined with bilateral anterior pelvic ring injury were treated with cannulated screw fixation alone (11 cases), INFIX fixation (19 cases), and “hybrid surgery” (13 cases). Among these patients, 21 with unsatisfactory reduction of displaced fractures underwent closed reduction or limited open reduction under anesthesia to restore the normal anatomic structures and to establish a bone tunnel for screw placements; 13 complex anterior pelvic ring injury patients with difficult reductions required “hybrid surgery.”

A total of 274 screws were inserted with robotic assistance, of which 262 screws were successfully inserted to a satisfactory position on the first attempt. The average number of screws inserted per patient was 3.2. The average operative time was 175 ± 32.6 min (range, 35–280 min). The average length of the incisions in which the screws were inserted was 2 cm. The average intraoperative blood loss during the robotic procedure was 35.2 ± 3.6 mL (range, 5–50 mL) and the average blood loss during hybrid surgery was 270 ± 156.7 mL (range, 50–650 mL). The screws inserted with robotic assistance were exposed to radiation an average of 29.1 ± 10.5 times (range, 9–63 times) during the surgery. The total fluoroscopy time was 6–42 s, and the average fluoroscopy time for each screw was 6.1 ± 0.2 s.

### *Surgical Complications*

There were 13 unsatisfactory intraoperative guiding needle placements, including 5 with obvious needle deviations that remained in the bone tunnel (re‐planning for screw placement was not performed), and 8 with cutting or penetration of the cortex.

As shown in Table 1, there were 2 cases of penetration of pubic cortex in the 32 posterior pelvic ring injury with unilateral anterior pelvic ring injury patients, among which 1 case resulted in hemorrhage of the “crown of death”. In that case, timely hemostasis was successfully achieved, and the operation was changed to a “hybrid surgery.” Among the 32 patients, there was 1 case of needle cutting and 1 case of penetration of the sacrum cortex. Among the 43 posterior pelvic ring injury with bilateral anterior pelvic ring injury patients, there was 1 case of needle cutting of the pubic ramus cortex and there were 2 cases of needle cutting of the sacrum cortex. One case of penetration through the iliac wing occurred among nine cases of (combined) acetabular fractures and/or iliac wing fractures. In the above 7 patients, satisfactory guiding needle insertions were successfully made after adjusting or re‐planning the trajectories.

In 1 posterior pelvic ring surgery, the guiding needle penetrated the sacral foramen and caused sacral nerve injury. In 1 open INFIX fixation, the lateral femoral cutaneous nerve was injured. Both patients’ injuries and symptoms resolved after treatment with neurotrophic drugs.

All wounds after robot‐assisted surgeries were healed by primary intention. After the “hybrid surgery” cases, there was 1 case of (subcutaneous) wound infection, 1 case of necrosis along the skin wound edges, and 1 case of ecchymosis. The ecchymosis disappeared spontaneously within 2 weeks, and the subcutaneous infection and skin necrosis resolved after changing the dressing.

### *Accuracy Evaluation*

Postoperative imaging revealed that the pelvic rings were in good shape. The CT scans showed that all sacroiliac screws inserted under robotic assistance were located within the boundary of the sacrum. There were no anterior or posterior edges observed on the sacrum, sacral canal, or sacral foramen. The pubic ramus screws were all located within the bone tunnel of the symphysis pubis with no penetration of the anterior–posterior or superior–inferior edges of the pubis. The positions of the sacroiliac screws were assessed as Lonstein^20^ Grade 0. At the last follow‐up, axial plane images of the screw placement positions planned intraoperatively using the robot and axial plane images of actual screw placement positions during surgery were imported to the Beyond Compare software to evaluate the accuracy of the robotic placements^21^. The positioning error was 2.31 ± 1.03 mm, and the angular error was 2.24° ± 1.32°.

### *Clinical Outcomes and Functional Evaluation*

All patients’ fractures healed within 3 months of surgery except for 2 patients with fixation failures who underwent secondary surgeries. One patient with combined posterior pelvic ring injury and symphysis pubis diastasis was treated with steel plate fixation of the anterior pelvic ring, but the fixation failed because of loosening of the screws. Secondary fixation using a steel plate was performed. In 1 case (symphysis pubis diastasis combined with vertical instability) of INFIX fixations, the symphysis pubis was separated again, and plate fixation was performed instead.

All robotic‐assisted screw insertions were neither loose, displaced, nor broken, and the patients’ postoperative lower limb functions were normal. The 19 patients who underwent anterior pelvic ring INFIX fixations tolerated the procedures without difficulty. Five patients with slim body types experienced local irritation from the pedicle screw tips, and the INFIX devices were removed after 12 weeks.

According to the Majeed score classification, 57 cases obtained excellent results, 26 good, and 3 fair. The average score was 92.4 points at the last follow‐up. The 3 fair cases were all severe injuries with poor reduction patients, included 1 case of hybrid surgery and 2 cases of INFIX fixation.

**Figure 1:**
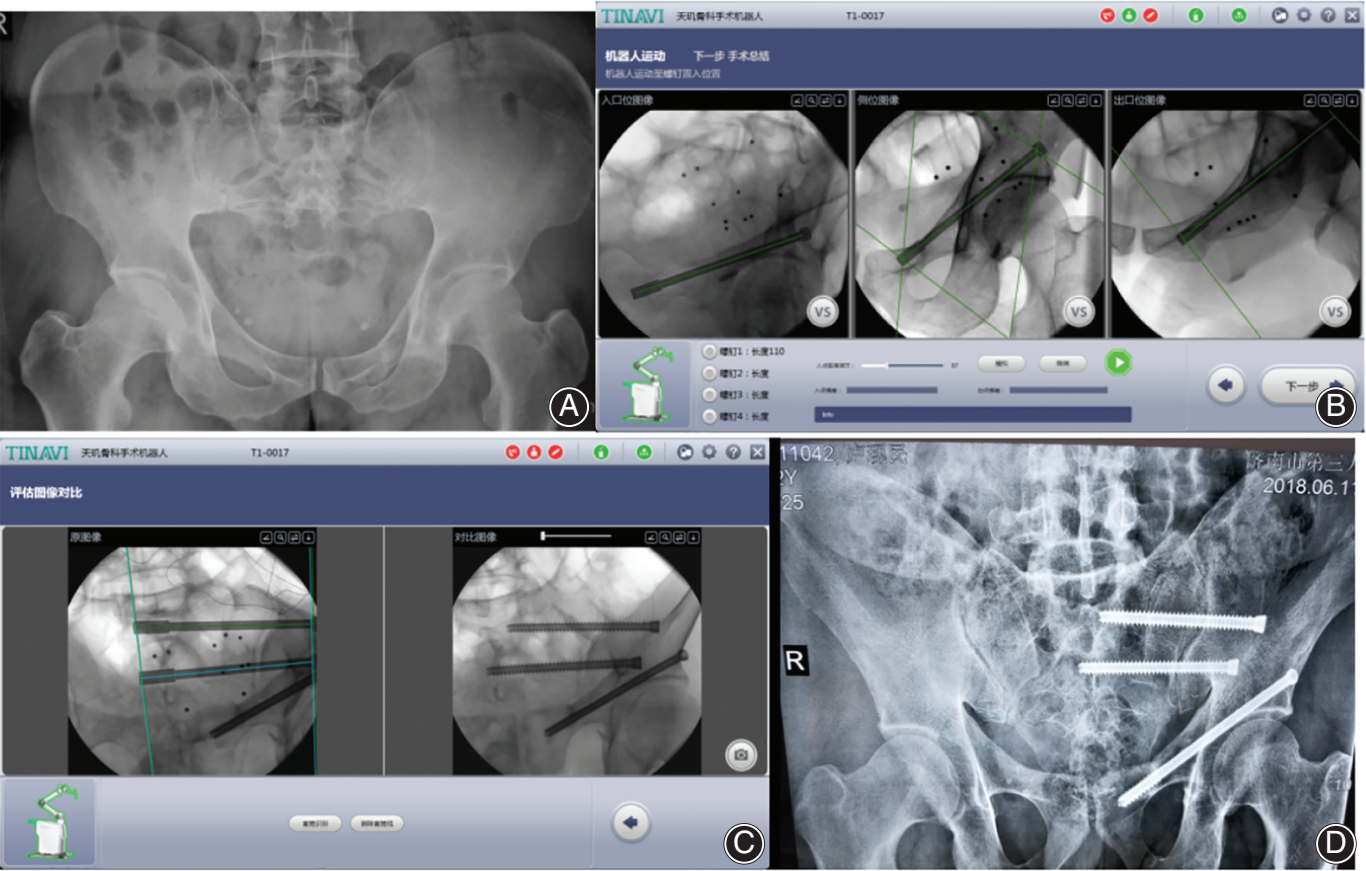
Male, 52 years old, height falling injury, left Dennis II sacrum fracture, and left ramus of pubis fracture, treated with robot‐assisted percutaneous cannulated sacroiliac screw fixation and pubic ramus screw fixation. (A) Preoperative X‐ray pelvic anteroposterior image. (B) Path planning of pubic ramus screw placement. (C) Path planning and screw placement of sacroiliac joint. (D) Postoperative follow‐up of pelvic anteroposterior image.

**Figure 2:**
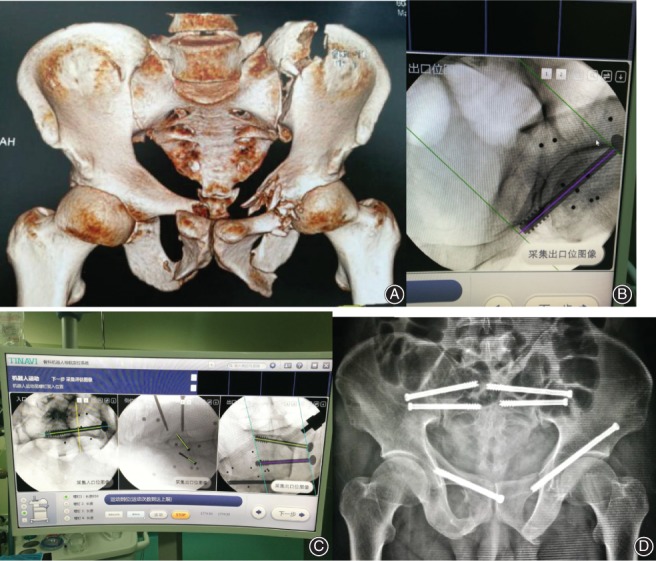
Male, 47 years old, car accident injury, bilateral iliac and pubic rami fractures, treated with robot‐assisted percutaneous cannulated sacroiliac screw fixation and pubic ramus screw fixation. (A) Preoperative CT 3D reconstruction. (B) Path planning of pubic ramus screw placement. (C) Path planning of sacroiliac screw placement. (D) Postoperative pelvic anteroposterior image.

**Figure 3:**
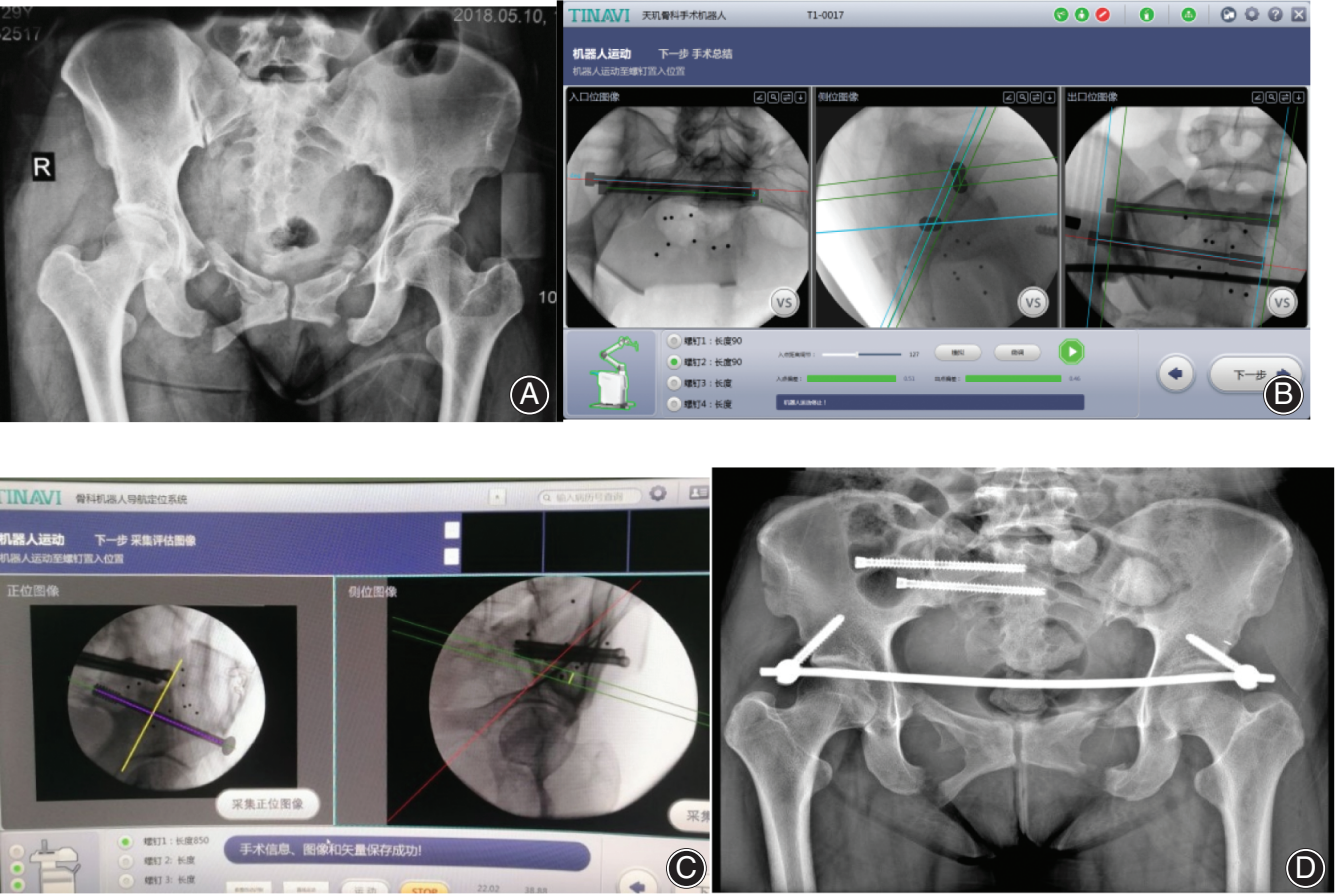
Female, 28 years old, crush injury, right sacrum fracture and bilateral iliac and pubic rami fractures, treated with robot‐assisted percutaneous cannulated sacroiliac screw fixation and robot‐assisted pedicle screw placement in the anterior inferior iliac spine along with INFIX fixation. (A) Preoperative X‐ray pelvic anteroposterior image. (B) Path planning of sacroiliac screw placement. (C) Path planning of pedicle screw placement in the anterior inferior iliac spine. (D) Postoperative pelvic anteroposterior image.

Typical cases are shown in Fig. 1, 2, 3.

## Discussion

Robot surgery is characterized by small trauma, quick recovery, and good technical, economic and social benefits. Precision medicine with robot technology at the core is the future development direction of orthopaedics. At present, in China, our center has carried out a relatively large number of robot‐assisted pelvic surgeries and accumulated some experience. Here, the authors discuss some of the issues faced in carrying out this operation.

### *Surgical Strategy for Robot‐assisted Pelvic Ring Injuries*

#### Isolated Anterior Pelvic ring (or Posterior Pelvic Ring) Injury

Robot‐assisted percutaneous cannulated screw fixation of the pubic ramus or robot‐assisted percutaneous cannulated screw fixation of the sacroiliac joint may be used in the management of isolated anterior or posterior pelvic ring injuries.

Isolated anterior pelvic ring injury is commonly seen in clinical practice. Straddle injuries can lead to anterior pelvic ring injuries. This study included 5 patients with isolated (unilateral or bilateral) anterior pelvic ring injuries. In principle, a conservative treatment approach can be adopted for anterior pelvic ring fractures without displacement or with only minor displacement. However, elderly patients, patients who cannot tolerate long‐term bed rest, and patients who require early movement can be actively treated using robot‐assisted minimally‐invasive percutaneous pubic ramus screw fixation according to the relative surgical indications. Intraoperative images of the obturator outlet, the pelvic inlet, and the pelvic anteroposterior view should be acquired to complete surgical planning, trajectory positioning, and screw insertions.

Isolated posterior pelvic ring injuries are rare in clinical practice. Posterior pelvic ring injuries should be managed with robot‐assisted sacroiliac screw fixations. Intraoperative images should be taken in the following order: pelvic inlet, pelvic outlet, and lateral view of the pelvis. Finally, the images should be used to guide trajectory planning and screw insertions.

#### *Combined Anterior and Posterior Pelvic Ring Injuries*

Combined anterior and posterior pelvic ring injury is the most common type of pelvic fracture, and is primarily characterized by unstable pelvic fractures that require a combined fixation of both the anterior and posterior pelvic rings. Combined anterior and posterior pelvic ring injuries can be classified into three types:

##### *Combined Posterior Pelvic Ring Injury and Symphysis Pubis Diastasis in the Anterior Pelvic Ring*

Robot‐assisted percutaneous cannulated screw fixation of the sacroiliac joint may be used to manage the posterior pelvic ring, and open screw or plate fixation of the symphysis pubis may be used to manage the anterior pelvic ring (hybrid surgery). For patients with symphysis pubis diastasis >2.5 cm, open reduction should be performed in the anterior pubis. Steel plate fixation is recommended to maintain reliable mechanical strength. Fixation of the anterior pelvic ring should be performed first, followed by fixation of the posterior pelvic ring.

##### *Combined Posterior Pelvic Ring Injury and Unilateral Anterior Pelvic Ring Injury*

Robot‐assisted percutaneous cannulated screw fixation of the sacroiliac joint may be used for the posterior pelvic ring, and robot‐assisted percutaneous cannulated screw fixation of the pubic ramus may be used for the anterior pelvic ring. The surgical order is irrelevant if the reduction is satisfactory.

##### *Combined Posterior Pelvic Ring Injury and Bilateral Anterior Pelvic Ring Injury*

This type of injury may be treated in three ways:

Cannulated screw fixation alone. Robot‐assisted (unilateral or bilateral) percutaneous cannulated screw fixation of the sacroiliac joint may be used for the posterior pelvic ring, and robot‐assisted (bilateral) percutaneous cannulated screw fixation of the pubic ramus may be used for the anterior pelvic ring. This approach is only suitable for fractures without obvious displacement or fractures that are easily reduced and have been treated successfully with screw fixation. Intraoperatively, the tracker should be alternately fixed for the trajectory planning of the bilateral pubic ramus and sacroiliac screws.

Combined cannulated screw fixation of the posterior pelvic ring and anterior pelvic ring INFIX fixation. This approach is more suitable for obese patients with “open book‐like injuries.” The integrity of the pelvic ring can be properly restored using a connecting rod. Therefore, fixation should be used to treat the posterior pelvic ring first, followed by the anterior pelvic ring. The INFIX pedicle screws can be placed manually or under robotic assistance. The latter involves acquiring LC‐2 front view and iliac oblique view images for trajectory planning, which is more accurate than freehand screw insertions and can reduce damage to the subcutaneous soft tissue. After INFIX fixation is performed in the anterior pelvic ring, the posterior pelvic ring can be treated using conventional robot‐assisted percutaneous cannulated screw fixation of the sacroiliac joint.

INFIX causes little trauma, has minimal impact on patients’ daily life, and is particularly useful in obese patients^22^. The authors believe that for patients in whom effective screw fixation of the anterior pelvic ring cannot be achieved, INFIX can be an effective and minimally‐invasive approach. The combined use of the TiRobot system and INFIX is a novel approach to minimally‐invasive treatment of complex combined anterior and posterior pelvic ring injuries^7^, ^23^.

“Hybrid surgery”: Combined robot‐assisted sacroiliac screw fixation of the posterior pelvic ring and open reduction and internal fixation of the anterior pelvic ring. Traditional open reduction surgery has many disadvantages, including the requirement for large incisions, significant stripping and damage to soft tissues, and bleeding. However, closed reductions are difficult to perform for complex “open book‐like injuries” and “mixed injuries.” Therefore, among patients with fractures in whom effective screw fixation cannot be achieved after open reduction, a traditional open reduction and internal fixation approach can be used as a last resort. The Stoppa approach or the ilio‐inguinal approach can be used.

(Combined) acetabular fractures and/or iliac wing fractures. Pelvic fractures often occur in combination with acetabular or iliac wing fractures. In principle, pelvic ring injuries require functional reduction and correction of alignment for relative stability, whereas acetabular fractures require anatomic reduction and absolute stability. In cases of combined acetabular fractures, patients with a relatively small total displacement of the articular surface that can be easily reduced may be treated with robot‐assisted minimally‐invasive cannulated screw fixation in the anterior column after reduction. Iliac wing fractures do not affect the pelvic ring and the requirement for stability is low. These fractures can be treated with robot‐assisted screw fixation or open reduction and internal fixation under direct vision. Even though complete anatomic reduction is not achieved, satisfactory postoperative clinical outcomes can be achieved.

### *Safety and Efficacy of Robot‐assisted Surgery for Pelvic Fractures*

The domestically‐developed orthopaedic surgery TiRobot from China uses a unique two‐plane positioning algorithm to complete the spatial positioning and adopts a modular design to facilitate the navigation of the surgical trajectory. The system is precise with respect to screw placement, with a positioning accuracy of 1 mm. Experimental research on robot‐assisted sacroiliac screw fixation^24^ and the increasing number of clinical studies^17^, ^18^ in China show that robotic surgery is precise, safe, and minimally invasive. It reduces radiation exposure time, allows for early postoperative activity, provides reliable fixation, and achieves satisfactory clinical outcomes.

The use of robots in orthopaedic surgery theoretically eliminates the instability introduced by surgeons during manual operations. Compared with open/manual surgery, the screws inserted via robotic assistance deviate less from the pre‐planned positions and also have lower needle penetration rates. In this study, a total of 274 screws were inserted under robotic assistance, of which 262 screws were successfully inserted into satisfactory positions on the first attempts. The success rate of one‐time screw placement was extremely high and the surgery was safe. Secondary injuries caused by deviation of the needle path and needle penetration occurred, which might be due to slippage of the needle tip, lateral stress, and surgeon error. However, robotic surgery has achieved unparalleled precision and safety compared to manual screw insertions.

### *Reduction and Screw Insertion Techniques*

A good reduction that restores the continuity and integrity of the bone tunnel is a prerequisite for screw placement. Patients with unstable combined anterior and posterior pelvic ring injuries who are also hemodynamically unstable prior to surgery (postadmission) should first be treated with external temporary fixations. Patients with vertical instability should be treated with lower extremity traction to correct the vertical displacement. Patients with unsatisfactory reductions can be treated with closed or open reductions. For pubic ramus fractures that are difficult to treat with closed reduction and those with displacements exceeding 10 mm, a small incision can be made using reduction forceps to reduce the fracture at its anterior end under fluoroscopic guidance until the reduction is satisfactory. Patients with sacral displacements >10 mm who do not show significant improvement after traction can be treated with posterior lumbo‐iliac screws and a rod to assist the reduction.

Currently, it is not feasible for a robot to complete all surgical procedures. The guiding needle and screw placements still need to be performed manually and cannot be monitored in real time. In addition, the screw placement process could injure the patient if the operators’ experience level is insufficient. To reduce subcutaneous damage and to avoid secondary damage caused by needle path deviation, the screw placement should be performed with adherence to the following: (i) the surgeon must be familiar with the patient's anatomy to avoid vascular and nerve damage, and repeated screw placement should be avoided to reduce damage to subcutaneous soft tissues; (ii) excessive traction should be avoided when opening channels in the soft tissue to reduce lateral stress; (iii) during sleeve insertion and as soon as the sleeve contacts the bone surface, the surgeon should pull in the opposite direction based on the needle insertion point to offset the gliding caused by the slanted bone surface; (iv) during guiding needle insertion, the electric drill should be reversed to avoid exerting excessive pressure on the bone surface, which is particularly useful when the guiding needle contacts the bone surface, as it can reduce gliding caused by the slanted bone surface; and (v) the surgeon must monitor the procedure attentively and act in a timely manner; if the cortex is penetrated, the needle should be withdrawn immediately and the direction should be adjusted with re‐planning of screw placement as needed to ensure the guiding needles and screws are located completely within the bone tunnel. Finally, these operations should only be performed by experienced surgeons or with experienced surgeons in attendance.

### *Precautions for Conducting Robotic Surgery and Existing Problems*

During robotic surgery, the operating environment should meet the regular working requirements specified in the robotic surgery manual. The operating room should be of suitable size with a good grounding system and power supply conditions. The operating table should meet the requirements for positioning and image acquisition. Surgeons in these cases must be experienced in the traditional surgical methods and skilled at closed reduction and internal fixation procedures. They must also possess the relevant anatomical knowledge to determine the accuracy of the navigation system and have the ability to switch to traditional surgery if there are hardware or software robotic system failures or sudden complications that preclude continuation of the robotic surgery. The surgeon must be familiar with the basic principles of the robotic system. They should complete robotic image acquisition accurately and plan the surgery based on scientific principles. This can ensure a smooth operation, reduce the “noninvasive time,” and shorten the overall operative time. For image acquisition, all bone structures in the operative area must be included, the bone tunnel should be continuous and complete, the calibrator should be clearly presented in fluoroscopic view, and the optical tracking camera should simultaneously recognize and capture the spatial position data from the patient tracker and the robot tracker. To avoid blurred images, the tracker position should be monitored closely during surgery, minimizing any factors that might interfere with the tracker, which will avoid loosening and shifting of the tracker and reduce error. The operation should be performed gently to avoid large relative displacements between the bone structures. Changes in the patient's position may cause changes in the spatial positions of the anatomic structures, resulting in a shift in the relative position between the patient and their tracker. If the angle between the guiding needle and the bone surface is too small, it could lead to incomplete fixation at the entry point and gliding of the needle tip, which will inevitably result in errors. Insertion of the guiding device and needles may deviate under excessive lateral stress if the sleeve is too long. Currently, this surgery cannot be performed under full robotic control. The surgeon is required to complete the screw placement trajectory planning according to the anatomic features and the fracture conditions using the software. Placement of the guiding needles and screws should be performed manually. Because it is not feasible to monitor the whole process, the surgeon must have a certain level of experience; however, subjective and operational errors may still occur.

### *Study Limitation*

This study is only a summary of the experience in our center without subdivision of the types of fracture injuries. At present, few centers can perform robot‐assisted pelvic surgery with an orthopaedic surgery robot, and no standards are available for robot‐assisted minimally‐invasive pelvic fracture treatment in China. For the next step, multi‐center controlled studies should be carried out, the strategies and skills of this surgery should be further investigated, and guidelines for robot‐assisted minimally‐invasive pelvic fracture treatment should be developed, so as to finally standardize the procedures and promote the popularity of this surgery.

## Conclusion

Most surgical techniques are being assessed from the perspective of performance using minimally‐invasive approaches. The application of the high‐end innovative robot, independently developed and made in China, for orthopaedic surgery provides a novel approach to the minimally‐invasive treatment of pelvic fractures. This study highlights an improvement in China's capability as a leader in the medical diagnosis and treatment of pelvic fractures. Rigorous selection of patients based on suitable indications, standardization of operating procedures, and assurance of surgical quality, along with appropriate surgical rationale and procedural efficacy, will surely allow the precise robotic approach to gain popularity and become widely adopted, which will benefit a vast number of orthopaedic patients.

## References

[1] Ward EF , Tomasin J , Vander Griend RA . Open reduction and internal fixation of vertical shear pelvic fractures. J Trauma, 1987, 27: 291–295. 3560270 10.1097/00005373-198703000-00011

[2] Stevenson AJ , Swartman B , Bucknill AT . Percutaneous internal fixation of pelvic fractures. German version. Unfallchirurg, 2016, 119: 825–834. 27659308 10.1007/s00113-016-0242-9

[3] Zhou KH , Luo CF , Chen N , Hu CF , Pan FG . Minimally invasive surgery under fluoro‐navigation for anterior pelvic ring fractures. Indian J Orthop, 2016, 50: 250–255. 27293284 10.4103/0019-5413.181791 PMC4885292

[4] Leung KS , Tang N , Cheung LW , Ng E . Image‐guided navigation in orthopaedic trauma. J Bone Joint Surg Br, 2010, 92: 1332–1337. 20884967 10.1302/0301-620X.92B10.24594

[5] Zheng G , Nolte LP . Computer‐assisted orthopedic surgery: current state and future perspective. Front Surg, 2015, 2: 66. 26779486 10.3389/fsurg.2015.00066 PMC4688391

[6] Karthik K , Colegate‐Stone T , Dasgupta P , *et al* Robotic surgery in trauma and orthopaedics: a systematic review. Bone Joint J, 2015, 97‐B: 292–299. 10.1302/0301-620X.97B3.35107 25737510

[7] Wang MY , Wang JQ . Medical robots and computer assisted navigation used in surgery of orthopaedic trauma. Chin J Orthop Trauma, 2005, 7: 1004–1009.

[8] Zwingmann J , Konrad G , Kotter E , Südkamp NP , Oberst M . Computer‐navigated iliosacral screw insertion reduces malposition rate and radiation exposure. Clin Orthop Relat Res, 2009, 467: 1833–1838. 19034594 10.1007/s11999-008-0632-6 PMC2690740

[9] Wong JM , Bewsher S , Yew J , *et al* Fluoroscopically assisted computer navigation enables accurate percutaneous screw placement for pelvic and acetabular fracture fixation. Injury, 2015, 46: 1064–1068. 25683211 10.1016/j.injury.2015.01.038

[10] Zhang W , Zhang LH , Tao S , *et al* Clinical effect and complication of minimally invasive surgery in treatment of anterior and posterior pelvic ring injury. Chin J Orthop Trauma, 2014, 30: 647–651.

[11] Ren MG , Luo CF , Chen J , *et al* Comparison between the C‐arm fluoroscopy and computer navigation in minimally invasive fixation with cannulated screws for pelvic fractures. Chin J Orthop Trauma, 2007, 9: 923–927.

[12] Giráldez‐Sánchez MA , Lázaro‐Gonzálvez Á , Martínez‐Reina J , *et al* Percutaneous iliosacral fixation in external rotational pelvic fractures. A biomechanical analysis. Injury, 2015, 6: 327–332. 10.1016/j.injury.2014.10.058 25554422

[13] Zhang L , Peng Y , Du C , *et al* Biomechanical study of four kinds of percutaneous screw fixation in in two types of unilateral sacroiliac joint dislocation: a finite element analysis. Injury, 2014, 45: 2055–2059. 25457345 10.1016/j.injury.2014.10.052

[14] Thakkar SC , Thakkar RS , Sirisreetreerux N , Carrino JA , Shafiq B , Hasenboehler EA . 2D versus 3D fluoroscopy‐based navigation in posterior pelvic fixation: review of the literature on current technology. Int J Comput Assist Radiol Surg, 2017, 12: 69–76. 27503119 10.1007/s11548-016-1465-5

[15] Matityahu A , Kahler D , Krettek C , *et al* Three‐dimensional navigation is more accurate than two‐dimensional navigation or conventional fluoroscopy for percutaneous sacroiliac screw fixation in the dysmorphic sacrum: a randomized multicenter study. J Orthop Trauma, 2014, 28: 707–710. 24694553 10.1097/BOT.0000000000000092

[16] Liu HS , Duan SJ , Jia FS , *et al* TiRobot‐assisted percutaneous screw fixation in unstable pelvic ring fractures. J Shandong Univ (HealthScience), 2017, 55: 103–109.

[17] Jiang KL , Tian W , Jia J . TiRobot surgical robotic navigation and location system assisted percutaneous iliosacral screws for treatment of unstable pelvic posterior ring injuries. J Tianjin Med Univ, 2017, 23: 247–251.

[18] Zhao CP , Wang JQ , Su YG , *et al* Clinical research on robot‐assisted percutaneous pelvic and acetabular screws surgery. J Peking Univ (Health Sciences), 2017, 49: 274–280. 28416838

[19] Liu HS , Duan SJ , Liu SD , Jia FS , Zhu LM , Liu MC . Robot‐assisted percutaneous screw placement combined with pelvic internal fixator for minimally invasive treatment of unstable pelvic ring fractures. Int J Med Robot, 2018 Jun 19, 14: e1927. 10.1002/rcs.1927 PMC6175104 29920914

[20] Deng N , Wu WJ , Liang GS . Robotic and computer assisted orthopaedic surgery. Chin J Orthop Trauma, 2005, 7: 620–624.

[21] Lonstein JE , Denis F , Perra JH , *et al* Complications associated with pedicle screws. J Bone Joint Surg Am, 1999, 81: 1519–1528. 10565643 10.2106/00004623-199911000-00003

[22] Vaidya R , Colen R , Vigdorchik J , Tonnos F , Sethi A . Treatment of unstable pelvic ring injuries with an internal anterior fixator and posterior fixation: initial clinical series. J Orthop Trauma, 2012, 26: 1–8. 22048183 10.1097/BOT.0b013e318233b8a7

[23] Liu HS , Duan SJ , Jia FS , *et al* Robot‐assisted transcutaneous screw combined with INFIX for unstable pelvic fracture: a case report. Chin J Orthop, 2017, 37: 1054–1056.

[24] Su YG , Wang JQ , Liu WY , *et al* The bi‐planar navigation robot system: application for insertion of sacroiliac screws. Chin J Orthop Trauma, 2006, 8: 45–49.

